# The Response of Fecal Microbiota and Host Metabolome in Dairy Cows Following Rumen Fluid Transplantation

**DOI:** 10.3389/fmicb.2022.940158

**Published:** 2022-07-13

**Authors:** Shuai Huang, Gang Zheng, Hongkai Men, Wei Wang, Shengli Li

**Affiliations:** ^1^College of Animal Science and Technology, Hainan University, Haikou, China; ^2^State Key Laboratory of Animal Nutrition, Beijing Engineering Technology Research Center of Raw Milk Quality and Safety Control, College of Animal Science and Technology, China Agricultural University, Beijing, China; ^3^Institute of Animal Sciences, Chinese Academy of Agricultural Sciences, Beijing, China

**Keywords:** rumen microbiota transplantation, fecal microbiota, serum metabolome, dairy cows, sequencing

## Abstract

Rumen fluid transplantation (RFT) has been used to rebuild rumen bacterial homeostasis, reshape rumen function, and restore rumen fermentation, whereas the effect of RFT on fecal microbiota and host metabolism in cows remains poorly understood. In our study, a combination of 16S rRNA sequencing and serum non-targeted metabolomics was performed to investigate the response of fecal microbiota and serum metabolome in dairy cows following RFT. Twenty-four prepartum dairy cows were randomly assigned to 3 groups (*n* = 8) for infusion of either saline (Con), fresh rumen fluid (FR), or sterilized rumen fluid (SR) after calving. Fourteen days after calving, fecal microbiota and serum metabolome were analyzed. The sequencing data of fecal samples revealed no changes in alpha diversity and relative abundance of dominant genera such as *Ruminococcaceae* UCG-005, *Rikenellaceae* RC9 gut and *Eubacterium coprostanoligenes*. However, the other genus level taxa, such as *Eubacterium oxidoreducens*, *Anaerorhabdus furcosa*, *Bacillus* and *Selenomonas*, showed distinct changes following RFT. Serum metabolome analysis showed that FR or SR infusion affected amino acids metabolism, bile acids metabolism and fatty acids metabolism (including linoleic acid, oleic acid and palmitic acid). Furthermore, correlation analysis showed that taxa from genera *Clostridiales* were positively correlated with metabolites involved in tryptophan and bile acid metabolisms, such as OTU1039 from genera unclassified *o_Clostridiales* was positively correlated to indoleacetic acid and taurolithocholic acid. These results suggest that RFT altered the composition of the fecal microbiota and modulated microbial metabolic pathways, which is vital for the development and safety assessment of rumen microbial intervention strategies.

## Introduction

The transition period is the most sensitive window for dairy cows due to dramatic changes in host physiology and nutrient metabolism further resulting in a higher incidence of metabolic disorders, mammary gland infections, and reproductive disorders (Allen and Piantoni, 2013). Moreover, a previous study revealed that the dramatic changes in physiology and nutritional factors may lead to dynamic changes in the structure of the rumen bacterial communities (Zhu et al., 2017). Recent studies of transition dairy cows demonstrated that rumen microbiota was shifted during the transition period (Lima et al., 2015; Zhu et al., 2018). Our previous work also confirmed that the fecal microbiota of dairy cows was changed during the freshening period (Huang et al., 2020). The gastrointestinal tract microbiota of cows plays a vital role in the forage and other plant materials digestion, volatile fatty acid, and microbial crude protein production (McCann et al., 2014). Additionally, previous studies have suggested that the gastrointestinal tract microbiota was associated with cows’ health (Mao et al., 2012; Plaizier et al., 2017) and productivity traits, such as feed intake (Li et al., 2020; Huang et al., 2021), milk production efficiency (Shabat et al., 2016; Weimer et al., 2017), milk yield (Indugu et al., 2017; Sofyan et al., 2019), and milk composition (Xue et al., 2020; Wu et al., 2021). Therefore, the transition period is a major challenge for cows’ productivity and health, farm profitability, and animal welfare.

Currently, a growing body of studies is being conducted regarding reshaping the gut microbiome to modulate metabolic disorders (Cheng et al., 2018; Depommier et al., 2019). In studies of humans and mice, fecal microbiota transplantation has been verified to be effective in reshaping the gut microbiota (Dodiya et al., 2019; Tian et al., 2020), repairing the intestinal barrier (Fischer et al., 2015), restoring intestinal function (De Palma et al., 2017), and changing behavior (Fujii et al., 2019; Hazan, 2020). In ruminants, rumen fluid or content transplanting was carried out to reshape the rumen microbiome and ruminal fermentation parameters, alleviate rumen epithelium damage (Liu et al., 2019), as well as recover the rumen homeostasis (Liu et al., 2019; Mu et al., 2021). In addition, Yu et al. (2020) revealed that rumen fluid oral inoculation affected the rumen microbiota and the colonic microbiota, and increased the relative abundance of *Veillonella*, *Ruminococcus* 1, *Anaerofilum*, and *Coprobacter* in weaning lambs. However, there is still a scarcity of knowledge available on the effect of RFT affects the fecal bacterial community and host metabolism of dairy cows.

A recent study in humans aroused our concern, they found that sterile filtrates from donor stool transplantation, rather than fecal microbiota, were sufficient to restore normal stool habits and eliminate symptoms in patients with *Clostridium difficile* infection (Ott et al., 2017). Another study found that pasteurized *Akkermansia muciniphila* can decrease liver dysfunction and inflammation-relevant blood markers levels with no significant difference in the gut microbiome (Depommier et al., 2019). Therefore, we hypothesized that fresh rumen fluid (FR) and sterile rumen fluid (SR) from donor cows may induce significant differences in fecal microbiota and host metabolism. To test this hypothesis, we used 16S rRNA gene sequencing and metabolomics to investigate the fecal bacteria and host metabolism variation following RFT. Our study presents some new insights into RFT regulation of gastrointestinal microbiota and host metabolism for dairy cows.

## Materials and Methods

### Experimental Design, Animals and Sample Collection

A total of twenty-four postpartum Holstein dairy cows with similar age (2–3-year-old) and body condition scores [3.38 ± 0.24, mean ± standard deviation (SD)] were randomly divided into three groups with 8 cows per group. The Con cows were dosing 10 L saline, the FR cows were dosing 10 L fresh rumen fluid and the SR group were dosing 10 L γ-ray sterilized rumen fluid daily from 1 to 3 days after calving, respectively. Rumen fluid was collected from twelve cannulated lactating Holstein cows (2–3-year-old, 59.67 ± 0.47 days in milk, 50.03 ± 0.42 kg/d milk yield, mean ± SD) via the rumen cannula and mixed in a sealed insulated container (37°C). One part of rumen fluid was immediately fed to FR cows, and another was immediately sent to the Hongyisifang irradiation company (Beijing, China) for sterilizing using γ-ray, and then fed to SR cows. The γ-ray condition was as follows: intensity 25 kGy, 10 h, 37°C. After irradiation, the bacterial load within the sterilized rumen fluid was found to be lower than 10 cfu/mL. Moreover, the bacterial number of the FR and SR group was 8.93 × 10^6^ and negative using RT-PCR, respectively. For transplanting, 12 people worked together and the entire transfer progress was finished within 15 min to minimize the negative effects of oxygen exposure on the rumen microbiota. All the selected animals were healthy and received no therapeutic or prophylactic antimicrobial treatment based on the veterinary records during the current lactation cycle or in the past 6 months.

Animals were housed in a free-stall barn of a commercial farm (Beijing, China) and had *ad libitum* access to fresh water. Cows were fed twice daily (07:00 and 14:30), and milked thrice daily (06:30, 14:00, and 22:30) for the first 4 days after calving and fed thrice daily (07:00, 14:30, and 20:30) and milked thrice daily (06:30, 15:00, and 22:30) for the remainder of the trial period. Milk yield was recorded daily by an automated milking machine (2 × 48, BouMatic Company, Madison, WI, United States). The donor cows were fed the early lactation diet for more than one month. The fresh cows were fed a fresh diet from calving to 14 days after calving. The nutritional composition and the chemical composition of the diets are present in Supplementary Table 1.

Fecal (*n* = 24) and blood samples (*n* = 24) were collected before morning feeding at 14 days after calving, respectively. Feces were collected by hand from the rectum using sterile gloves, immediately placed on wet ice and further frozen at –80°C until DNA extraction. Blood was collected through the tail vein, stratified at room temperature for 30 min and subsequently centrifuged (4°C) at 3,000 *g* for 15 min. Serum samples were obtained from the supernatant and were stored at –80°C until further analysis, respectively.

### DNA Extraction and Amplicon Sequencing

Total DNA of fecal samples was extracted using an OMEGA Stool DNA kit (Omega Bio-Tek, Norcross, GA, United States) according to the manufacturer’s protocols. The concentration and quality of DNA were evaluated using a NanoDrop 2000 spectrophotometer (NanoDrop Technologies, Wilmington, DE, United States) and 1% agarose gel. Amplification of the V3 to V4 regions of the 16S rRNA gene was performed using 338F (5′-ACTCCTACGGGAGGCAGCAG-3′) and 806R (5′-GGACTACNNGGGTATCTAAT-3′) primers (Ren et al., 2017), with an 8-base sequence barcode for each sample. The PCR mixture contained 12.5 mL KAPA 2G Robust Hot Start Ready Mix (Kapa Biosystems, Wilmington, MA, United States), 1 μl of each primer (5 mM), 5 μl template DNA (6 ng/μl) and 5.5 μl ddH_2_O. The PCR conditions were performed as follows: 5 min at 95°C for initial denaturation, 28 cycles of 45 s at 95°C, 50 s at 55°C, 45 s at 72°C, then 10 min at 72°C for the final extension. The PCR products were quantified by 1% agarose gel and purified by Agencourt AMPure XP Kit (Beckman Colter Genomics, Indianapolis, IN, United States). Purified products were sequenced (paired-end, 2 × 250 bp reads) on Illumina’s MiSeq PE300 platform (Illumina, San Diego, CA, United States) following the standard Illumina sequencing protocols (Caporaso et al., 2012).

### Sequence Analysis

Sequence reads from the Illumina MiSeq platform were analyzed using QIIME 1.8.0 (Caporaso et al., 2010), and bases with an average quality score > 20 and long reads > 200 bp were retained. Paired-end reads were merged into tags using FLASH, with a minimum overlap of a 10-base sequence. Chimeric sequences were identified and removed using the UCHIME algorithm (Edgar, 2010), and the remaining sequences were clustered into the operational taxonomic unit (OTU) at a 97% similarity threshold using the Ribosomal Database Project classifier (Cole et al., 2009). The SILVA database was used for taxonomic characterization (SILVA Release 123, July 2015, Bremen, Germany; Quast et al., 2013). The representative OTU table was obtained after all singletons and doubleton OTUs removing using UCLUST (Schloss, 2009). All samples were normalized by subsampling to 38,317 sequences, the size of the smallest sample. The rarefaction curve and bar graphs were constructed to assess the sufficient sequencing depth (Supplementary Figure 1). The normalized counts of OTU by sample were used for further analysis of α diversity, β diversity, and taxonomic classification. Alpha diversity indices were calculated using mothur 1.30.1, with the Chao1 index to calculate species richness and Shannon index to calculate species diversity (Schloss, 2009). Beta diversity of the samples was calculated based on weighted and unweighted UniFrac distance and visualized by a principal coordinate analysis (PCoA) plot with the R package “GUniFrac” and “ape” (Chen et al., 2012; Paradis and Schliep, 2018). Statistical significance of the bacterial composition community was analyzed by non-parametric PERMANOVA after 999 random permutations using the “vegan” package in R (Oksanen et al., 2015).

### Serum Metabolomics

After thawing, 100 μL serum was mixed with 400 μL of cold methanol/acetonitrile (1:1, v/v). The mixture was vortexed for 1 min, sonicated twice at 4°C for 30 min, incubated at –20°C for 1 h to precipitate protein, and then centrifuged for 20 min at 14,000 *g*. The supernatant was collected, transferred to a microtube, dried at –80 °C, and then resuspended in 100 μL acetonitrile/water (1:1, v/v) solvent before LC-MS/MS analysis. Each serum sample (10 μL) was mixed and used as a quality control (QC) sample. Before injecting the samples, the instrument was stabilized using three QC samples, and then, the QC samples were run once after every 12 samples for monitoring the stability and repeatability of the instrument.

Serum metabolome was performed using an Agilent 1290 liquid chromatography system (Agilent Technologies, Santa Clara, CA, United States). Two microliters of the sample were injected into an Acquity BEH Amide column (1.7 μm, 2.1 mm × 100 mm, Waters, Milford, MA, United States) under a flow rate of 0.3 mL/min. The mobile phase consisted of 25 mmol/L ammonium acetate and 25 mmol/L ammonium hydroxide in water (mobile phase A) and acetonitrile (mobile phase B). The gradient elution was performed as follows: 0–0.5 min, 95% B; 0.5–7 min, 95–65% B; 7–8 min, 65–40% B; 8–9 min, 40–95% B; 9–11.9 min, 95% B. The separated components were detected using an Agilent 6545 quadruple time-of-flight (Q-TOF) mass spectrometer (Agilent Technologies, Santa Clara, CA, United States) equipped with an electrospray ionization source. The ion spray voltage was set at 250 V in the positive mode and 1.5 kV in the negative mode. Both the gas and sheath temperatures were maintained at 650°C. The ion source gas 1 was set at 40 psi and gas 2 was set at 60 psi.

### Metabolomic Data Preprocessing and Multivariate Statistics

All raw data files were converted to mzXML format using ProteoWizard software 3.0.8^1^. The XCMS program was applied for peak extraction, aligning, retention time correction and peak area calculating. A support vector regression method was used to correct filtered peaks. All detected ions were normalized based on the relative intensity between the area of the detected feature and the area of the QC samples [equation: area(feature)/area (QC)]. The normalized data involving the peak number, sample name, and peak area were imported into the SIMCA software package 14.1 (Umetrics AB, Umea, Sweden) for univariate statistical analysis (Student’s *t*-test) and multivariate statistical analysis [including principal component analysis (PCA) and projections to latent structures discriminant analysis (PLS-DA)]. PLS-DA was used to obtain maximal covariance between measured data and response variables. The metabolites were identified and validated by the LECO-Fiehn Rtx5 database (Kind et al., 2009) and public databases, that is, KEGG^2^ and NIST^3^. The screening criteria for differential metabolites were identified according to variable importance in projection (VIP) > 1 (generated by the PLS-DA model) and *P* < 0.05 (from univariate statistical analysis). KEGG pathway analysis of differential metabolites was carried out using MetaboAnalyst 5.0^4^ according to the *Bos taurus* database. All the matched pathways, according to the *P* values from the pathway analysis and pathway impact values from the pathway topology analysis, are shown in the metabolome map. The pathways with both FDR values > 0.1 and *P* values < 0.05 were regarded as key pathways.

### Statistical Analysis

The difference in bacterial richness, diversity and the relative abundance of bacteria at phylum and genus levels was performed using the R package ‘‘dplyr’’^5^ with Kruskal-Wallis for multiple comparisons and Wilcoxon rank-sum test for pairwise comparisons. The OTU table was transformed using variance stabilizing transformation to account for differences in library size and significant log2 fold differences between treatments were determined by DESeq2 in R (Love et al., 2014). All *P* values were done the false discovery rate correction, as described by Benjamini and Hochberg (1995). The corrected *P* value ≤ 0.05 were considered as the signifcant diference.

Spearman’s correlation of the different abundance OTUs (average relative abundance > 0.01%, *P* < 0.05) against significantly differential serum metabolites (VIP > 1.0, *P* < 0.05) was analyzed using the R package ‘‘Psych’’^6^. Only significant correlations (| r_s_| ≥ 0.41, *P* < 0.05) were presented and further visualized using the R package “pheatmap”, where r_s_ is defined as the Spearman rank-order correlation coefficient.

## Results

### Effects of Rumen Fluid Transplantation (RFT) on Fecal Bacterial Diversity and Structure

We sought to know what happened in the fecal microbiota of dairy cows after RFT and to understand how the RFT affects the fecal microbiome. After size filtering, quality control, and chimera removal, a total of 3,452,375 raw reads were generated through amplicon sequencing, with an average number of 138,095 ± 13,266 [mean ± standard error (SEM)] reads per sample. A total of 34,366 OTUs were detected within all samples, with an average of 1375 ± 26 OTUs per sample, which were affiliated with 17 phyla and 234 genera. Rarefaction curves showed that the number of newly identified OTUs was not increased by the number of sequences per sample (Supplementary Figure 1), implying the sampling depth was adequate to cover the fecal bacterial composition in the present study. The Good’s coverage estimates averaged more than 99.03%, implying that the current sequencing depth was sufficient to be representative of the microbiota studied.

**FIGURE 1:**
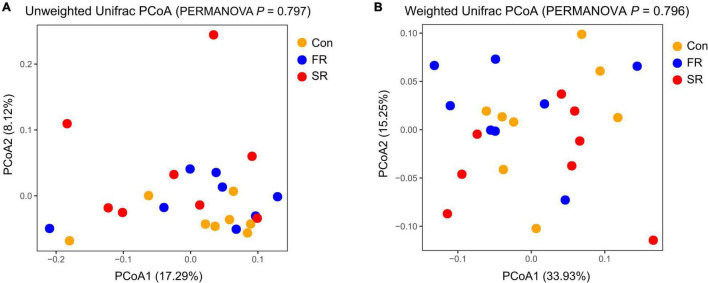
Principal Coordinate Analysis (PCoA) of fecal microbial community based on unweighted **(A)** and weighted Unifrac distance **(B)**. PERMANOVA anaysis with 999 permutations is shown. Con, saline; FR, fresh rumen fluid; SR, sterilized rumen fluid.

When examining community structure, there isno significant difference in the Chao1 and Shannon index between every two groups (*P* > 0.05, Supplementary Figure 2). The unweighted and weighted unifrac distance-based principal coordinates results showed that the microbial structure profiles did not significantly affect by the treatment (Figures 1A,B; PERMANOVA test, *P* = 0.797 and *P* = 0.796).

### Effects of Rumen Fluid Transplantation (RFT) on Fecal Bacterial Composition

At the phylum level, the predominant phyla, whose proportions were > 1%, comprised 95 to 98% of the total phyla, including *Firmicutes*, *Bacteroidetes*, *Actinobacteria*, and *Spirochaetae* (Supplementary Figure 3A). The difference of fecal bacteria at the phylum and genus level was present in Table 1. The relative abundance of the predominant phyla was not significantly changed by FR or SR infusion (*P* < 0.05), except for *Fibrobacteres* (*P* = 0.032), which was significantly decreased by SR compared to the Con group. At the genus level, 15 abundant genera exhibited proportions > 1% (Supplementary Figure 3B). Among them, the SR group had a lower proportion of *Ruminococcaceae* UCG-013, *Blautia*, and *Fibrobacter* and a higher proportion of *Eubacterium rectale* and *Butyricicoccus* compared with the Con cows (*P* < 0.05). An increased proportion of *Pseudoramibacter*, *Quinella* and *Barnesiella*, and a decreased proportion of *Eubacterium rectale*, *Eubacterium oxidoreducens*, *Anaerorhabdus furcosa* and *Selenomonas* were observed in the FR cows compared to the Con cows (*P* < 0.05). Otherwise, some bacteria showed a significant difference between the FR and SR group, including *Eubacterium rectale*, *Lachnospiraceae* NK4A136, *Eisenbergiella*, and *Bacillus* (*P* < 0.05).

**TABLE 1:** Effects of rumen fluid transplantation on fecal microbiota at the phylum and genus level (the relative abundance > 0.01% of all samples).

Phylum/Genera	Group			SEM	*P*-value
	Con	FR	SR		
***Firmicutes***	**67.23**	**63.28**	**67.63**	**0.910**	**0.141**
*Ruminococcaceae* UCG-013	2.97^a^	2.71^ab^	2.18^b^	0.193	0.172
*Blautia*	0.26^a^	0.19^ab^	0.16^b^	0.017	0.057
*Eubacterium rectale*	0.11^b^	0.07*^c^*	0.18^a^	0.019	0.069
*Eubacterium oxidoreducens*	0.06^a^	0.03^b^	0.04^ab^	0.004	0.013
*Lachnospiraceae* NK4A136	0.03^ab^	0.06^a^	0.02^b^	0.011	0.112
*Eisenbergiella*	0.04^ab^	0.02^b^	0.04^a^	0.004	0.075
*Anaerorhabdus furcosa*	0.02^a^	0.01^b^	0.02^ab^	0.002	0.020
*Pseudoramibacter*	0.00^b^	0.04^a^	0.003^ab^	0.015	0.093
*Quinella*	0.002^b^	0.03^a^	0.007^ab^	0.006	0.054
*Butyricicoccus*	0.005^b^	0.01^ab^	0.02^a^	0.003	0.025
*Bacillus*	0.01^ab^	0.003^b^	0.02^a^	0.003	0.026
*Selenomonas*	0.02^a^	0.004^b^	0.01^ab^	0.003	0.036
***Bacteroidetes***	**26.32**	**28.29**	**26.26**	**0.974**	**0.756**
*Barnesiella*	0.17^b^	0.24^a^	0.20^ab^	0.014	0.106
***Fibrobacteres***	**0.12^a^**	**0.10^ab^**	**0.01^b^**	**0.019**	**0.032**
*Fibrobacter*	0.12^a^	0.10^ab^	0.01^b^	0.019	0.032

*a and b letters mean P < 0.05 between each two treatments.*

*SEM, the standard error of mean; Con, saline; FR, fresh rumen fluid; SR, sterilized rumen fluid.*

*Bold words and values mean the phylum level.*

**FIGURE 2:**
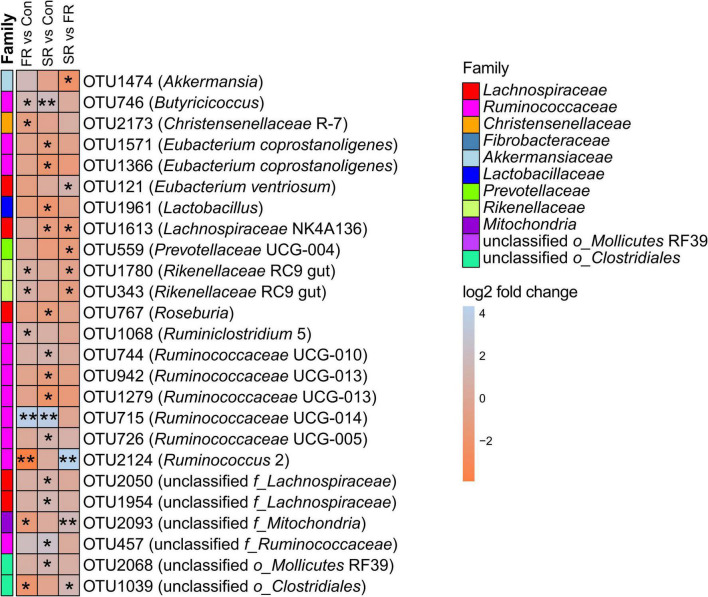
The significantly different OTUs were present in the heatmap. The OTUs was performed stabilizing transformation (log2 fold change) and pairwised comparisons using DESeq2. Con = saline, FR = fresh rumen fluid, SR = sterilized rumen fluid. *P < 0.05, **P < 0.01.

At the OTU level, a total of 2733 OTUs were detected in all fecal samples. Among them, 25 OTUs (the counts > 0.01% of total counts) showed the difference after RFT (*P* < 0.05, Figure 2). The SR group showed a higher proportion of OTUs from genera of *Ruminococcaceae* UCG-005 (OTU726), *Ruminococcaceae* UCG-010 (OTU744), unclassified *f_Lachnospiraceae* (OTU1954 and OTU2050), and unclassified *o_Mollicutes* RF39 (OTU2068); however, they showed a reduced proportion of OTUs from genera *Eubacterium coprostanoligenes* (OTU1571 and OTU1366), *Prevotellaceae* UCG-004 (OTU559), *Ruminococcaceae* UCG-013 (OTU942 and OTU1279), *Lachnospiraceae* NK4A136 (OTU1613), and unclassified *o_Clostridiales* (OTU1039) compared with the Con cows (*P* < 0.05). Similar, the FR cows showed a higher proportion of OTUs from genera *Ruminococcaceae* UCG-014 (OTU715) and *Butyricicoccus* (OTU746), and a lower proportion of OTUs from genera *Christensenellaceae* R-7 (OTU2173), unclassified *o_Clostridiales* (OTU1039), and *Ruminococcus* 2 (OTU2124) compared to the Con cows (*P* < 0.05). In addition, a total of 8 OTUs significantly differed between the FR and SR groups (*P* < 0.05), with 2 OTUs were in the family *Lachnospiraceae* (OTU121 and OTU1613), 2 in the family *Rikenellaceae* (OTU343 and OTU1780), 1 in the family *Prevotellaceae* (OTU559), 1 on the family *Ruminococcaceae* (OTU2124), 1 in the family *Akkermansiaceae* (OTU1474) and 1 in the family *Mitochondria* (OTU2093).

### Effects of Rumen Fluid Transplantation (RFT) on Serum Metabolome and Metabolic Pathway

To further investigate whether the FR or SR infusion affected the metabolite profiles in host metabolism, the LC-MS was performed to analyze the serum metabolite profiles. The parameters R_2_Y and Q_2_ were 1 and 0.964 in the positive ion mode, respectively, while they were 1 and 0.984 in the negative ion mode, respectively, indicating that the PLS-DA models were successfully established and had a satisfactory interpretative ability and predictive ability. An obvious separation between the Con and FR group, the Con and SR group, and the FR and SR group was observed in PLS-DA plots (Supplementary Figure 4), indicating that FR and SR could effectively alter the metabolic profiles of cows, and FR and SR play a different role in host metabolism.

A total of 306 compounds were identified in the serum metabolome. Based on the screening criteria and univariate statistical analysis (VIP ≥ 1, *P* value < 0.05), there are a total of 17, 14, and 5 metabolites that significantly differed between the Con and FR group, the Con and SR group, the FR and SR group, respectively (Table 2). Compared with the Con cows, a lower relative concentration of methionine, IAA, stearic acid, nervonic acid and oleic acid was observed both in FR and SR cows. Intriguingly, the relative concentration of bile acids, including glycocholic acid (GCA), glycodeoxycholic acid (GDCA), taurochenodeoxycholate (TCDCA), taurolithocholic acid (TLCA) and tauroursodeoxycholic (TUDCA), were significantly lower in FR cows than those in Con cows (*P* < 0.05). In addition, we found that both FR and SR decreased the tryptophan-derived metabolites (*P* < 0.05), including indoleacetic acid (IAA) and trimethylamine N-oxide (TMAO). Overall, these findings suggest that the host metabolism was affected after RFT. Compared to the FR group, the relative concentration of methionine, serine, salicylic acid and IAA were significantly lower in the SR group (*P* < 0.05), while myristic acid was significantly higher in the SR group (*P* < 0.05).

**TABLE 2:** The significantly different serum metabolites between saline (Con) and fresh rumen fluid (FR), saline (Con) and sterilized rumen fluid (SR) cows.

Superclass	Metabolites	FR vs. Con		SR vs. Con		SR vs. FR	
		FC^1^	*P*-value	FC^1^	*P*-value	FC^1^	*P*-value
Amino acid	L-Arginine	0.76	0.013	0.47	0.003		
	L-Methionine	0.82	0.006	0.78	0.034	0.68	0.009
	L-Proline	0.46	0.006				
	L-Glutamine	0.68	0.010				
	L-Serine			0.81	0.015	0.73	0.005
	N-Acetylornithine			0.77	0.034		
Tryptophan and derivatives	IAA	0.62	0.005	0.64	0.019	0.64	0.030
	Tryptophan	0.84	0.032	0.72	0.008		
	TMAO	0.80	<0.001				
Bile acids	GCA	0.56	0.006				
	GCDCA	0.67	0.012				
	TCDCA	0.64	0.007				
	TLCA	0.56	0.012				
	TUDCA	0.53	0.010				
Lipids	Stearic acid	0.71	0.041	0.64	0.010		
	Nervonic acid	0.66	0.044	0.61	0.009		
	Salicylic acid			0.61	0.016	0.44	0.014
	Myristic acid			1.29	0.036	1.50	0.022
Fatty acids	Linoleic acid	0.63	0.029	0.74	0.030		
	Oleic acid	0.57	0.010	0.68	0.028		
	Palmitic acid	0.74	0.018	0.87	0.004		
Energy	Oxoglutaric acid			1.29	0.029		

*^1^FC, fold change; IAA, indoleacetic acid; TMAO, trimethylamine N-oxide; GCA, glycocholic acid; GDCA, glycodeoxycholic acid; TCDCA, taurochenodeoxycholate; TLCA, taurolithocholic acid; TUDCA,; tauroursodeoxycholic; Con, saline; FR, fresh rumen fluid; SR, sterilized rumen fluid.*

**FIGURE 3:**
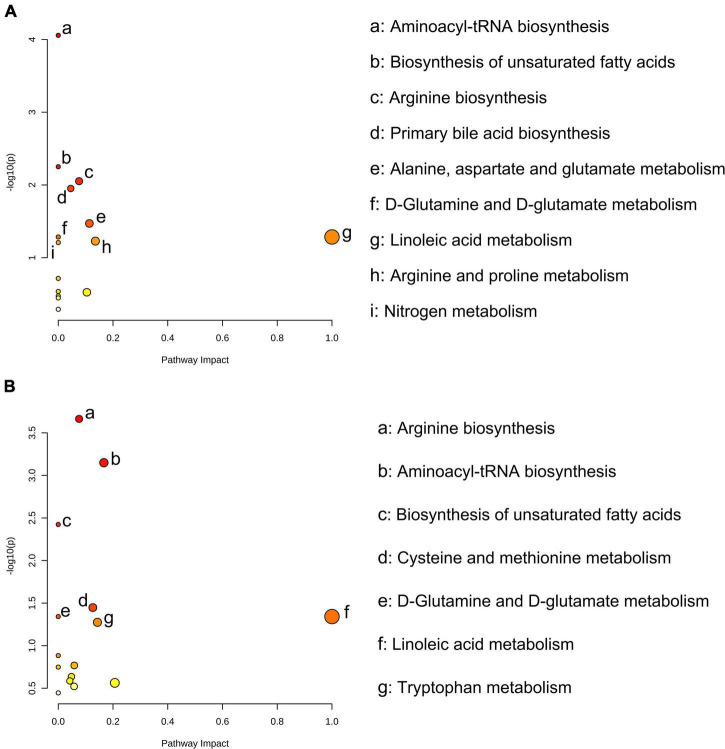
Significantly different serum metabolites between fresh rumen fluid (FR) and saline (Con) cows, and sterilized rumen fluid (SR) and Con cows. Pathway analysis was performed using the significantly different serum metabolites between FR and Con cows **(A)**, and SR and Con cows **(B)**. Con, saline; FR, fresh rumen fluid; SR, sterilized rumen fluid.

For the numerous differential metabolites, further exploration of the corresponding metabolic pathways that participated in the intervention was necessary. As shown in Figure 3A, the enrichment analysis of metabolic pathways of the differential metabolites suggested that biosynthesis of unsaturated fatty acids (*P* = 0.006), arginine biosynthesis (*P* = 0.009), primary bile acid biosynthesis (*P* = 0.011), alanine, aspartate and glutamate metabolism (*P* = 0.034) were the main metabolic pathways affected by FR intervention. Compared to Con group, SR significantly affected the biosynthesis of unsaturated fatty acids (*P* = 0.004), cysteine and methionine metabolism (*P* = 0.036), D-Glutamine and D-glutamate metabolism (*P* = 0.046) and linoleic acid metabolism (*P* = 0.046, Figure 3B).

### Correlation Between Fecal Bacteria and Different Serum Metabolites

**FIGURE 4:**
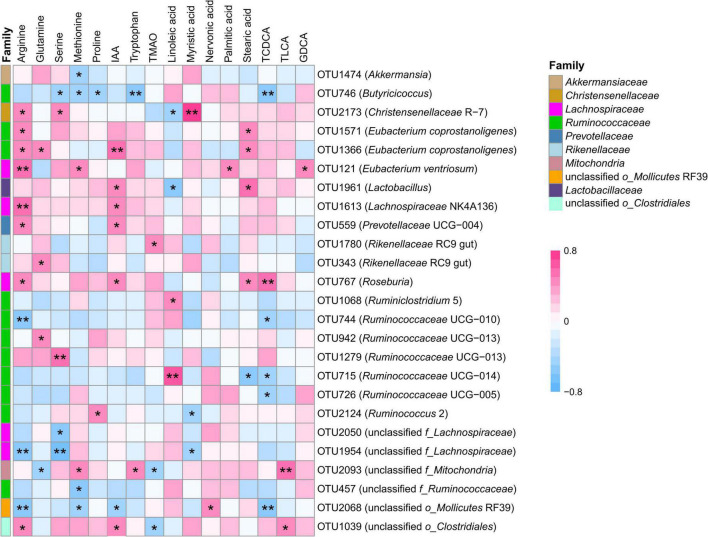
Correlation between transplantation-sensitive operational taxonomic units (significantly differed OTUs) and serum metabolites following rumen fluid transplantation. Red represents a positive correlation, and blue represents a negative correlation. Only strong (Spearman’s |r| > 0.40) and significant (*P* < 0.05) correlations are presented in the figure. *P < 0.05, **P < 0.01.

To determine the potential relationship between fecal microbiota and host metabolism, the Spearman’s correlation analysis was performed to detect whether and how fecal microbiota be attributed to metabolism (Figure 4). The results indicated that the relative concentration of tryptophan was positively correlated with the relative abundance of OTUs from genus unclassified *f_Mitochondria* (OTU2093, r_s_ = 0.42, *P* = 0.040), and negatively correlated to the relative abundance of *Butyricicoccus* (OTU746, r_s_ = –0.54, *P* = 0.007). The relative concentration of IAA was positively correlated to the relative abundance of bacteria within family *Lactobacillaceae*, including OTU764 (*Roseburia*, r_s_ = 0.41, *P* = 0.046), OTU1613 (*Lachnospiraceae* NK4A136, r_s_ = 0.45, *P* = 0.026) and OTU1961 (*Lactobacillus*, r_s_ = 0.46, *P* = 0.025) and family unclassified *o_Clostridiales* (OTU1039, r_s_ = 0.42, *P* = 0.041), while negatively correlated to the relative abundance of bacteria within genera unclassified *o_Mollicutes* RF39 (OTU2068, r_s_ = –0.48, *P* = 0.019). The relative concentration of TMAO was strongly correlated to 2 OTUs representing 2 genera, including *Rikenellaceae* RC9 gut (OTU1780, r_s_ = 0.45, *P* = 0.029), and unclassified *f_Mitochondria* (OTU2093, r_s_ = –0.41, *P* = 0.048). The relative concentration of methionine was positively correlated with the relative abundance of OTU121 within genera *Eubacterium ventriosum* (r_s_ = 0.48, *P* = 0.017), and negatively correlated with the relative abundance of OTU746 within genera *Butyricicoccus* (r_s_ = –0.46, *P* = 0.024). The relative abundance of OTU1633 from genera *Eubacterium coprostanoligenes* was positively correlated with the relative concentration of arginine (r_s_ = 0.45, *P* = 0.026) and glutamine (r_s_ = 0.42, *P* = 0.041). A positive correlation was observed between the relative concentration of palmitic acid and the relative abundance of OTUs from genera *Eubacterium ventriosum* (OTU121, r_s_ = 0.45, *P* = 0.026). The relative concentration of myristic acid was positively associated with the relative abundance of OTU2173 from genera *Christensenellaceae* R-7 (r_s_ = 0.80, *P* < 0.001), while this OTU was negatively associated with the relative concentration of linoleic acid (r_s_ = –0.46, *P* = 0.023). The relative concentration of linoleic acid was strongly correlated with the bacteria from family *Ruminococcaceae*, including OTU1068 (*Ruminiclostridium* 5, r_s_ = 0.49, *P* = 0.015), and OTU715 (*Ruminococcaceae* UCG-014, r_s_ = 0.62, *P* = 0.001). The relative concentration of nervonic acid was positively correlated to the relative abundance of OTU2068 from genera unclassified *o_Mollicutes* RF39 (r_s_ = 0.46, *P* = 0.024). OTU715 from genera *Ruminococcaceae* UCG-014 was negatively correlated with the relative concentration of stearic acid (r_s_ = –0.57, *P* = 0.004). For bile acids, a positive correlation was observed between the relative concentration of TLCA and the relative abundance of OTU1039 (unclassified *o_Clostridiales*, r_s_ = 0.47, *P* = 0.020) and OTU2093 (unclassified *f_Mitochondria*, r_s_ = 0.56, *P* = 0.005). A positive correlation was observed between OTU121 (*Eubacterium ventriosum*) and GDCA (r_s_ = 0.45, *P* = 0.026). The relative concentration of TCDCA was negatively correlated with the relative abundance of 5 OTU from genus *Ruminococcaceae* UCG-010 (OTU744, r_s_ = –0.42, *P* = 0.042), *Ruminococcaceae* UCG-005 (OTU726, r_s_ = –0.44, *P* = 0.033), *Ruminococcaceae* UCG-014 (OTU715, r_s_ = –0.44, *P* = 0.033), *Butyricicoccus* (OTU746, r_s_ = –0.53, *P* = 0.007), and unclassified *o_Mollicutes* RF39 (OTU2068, r_s_ = –0.55, *P* = 0.006), and positively correlated with the relative abundance of genus *Roseburia* (OTU767, r_s_ = 0.59, *P* = 0.005).

## Discussion

Manipulating the fecal microbiota is a promising approach for changing metabolism (Depommier et al., 2019; Gu et al., 2019), rebuilding microbial function (Ridaura et al., 2013), and improving farm animals’ traits (Li et al., 2019). Previous work in ruminants demonstrated that rumen fluid transplanting altered the rumen bacterial community (Mu et al., 2021; Yin et al., 2021) and ruminal fermentation (Liu et al., 2019). However, we still poorly understood what happened in the fecal microbiota and metabolism of cows following RFT. Hence, the goal of this work was to investigate the influence of RFT on fecal bacterial communities and serum metabolomics in fresh cows.

In the current study, we found that FR and SR induced no statistical difference in richness, diversity and bacterial community structures of fecal microbiota, but changed the relative abundance of some bacteria. This observation was similar to an earlier work, which reported that the rumen fluid infusion before and during weaning had no significant effect on the alpha and beta diversity of colon content bacterial communities in lambs, but some bacterial genera of colon content were affected by the inoculation (Yu et al., 2020). One possible reason for the difference in some fecal bacteria between FR and SR cows is the native competition. The SR cows contain dead microbes, fermentation products, and other metabolites, which provide more fermentation substrate to fecal bacteria. Although FR contains similar fermentation substrates to SR, FR had viable microbes, which compete for sites with the native (autochthonous) microbes. A previous study found that the native microbes are sufficiently well adapted in their native habitat to outcompete non-native strains or species introduced from other habitats, including other rumens (Weimer, 2015). Thus, we observed a lower number of differential fecal bacteria between the FR and Con group.

Moreover, we observed a lower abundance of pathogenic bacteria – *Eubacterium ventriosum* and benefical bacteria - *Butyricicoccus* after RFT, which could result in better hindgut functionality. *Eubacterium ventriosum* was correlated with obesity in children (Murugesan et al., 2015) and Japanese (Kasai et al., 2015). *Butyricicoccus* was a butyrate produce bacteria in the gut, which is critical to maintaining host gut health (Louis and Flint, 2017). Moreover, FR and SR increased the relative abundance of butyrate-producing bacteria *Ruminiclostridium* 5 and *Ruminococcaceae* UCG-014. These bacteria are able to produce butyrate by degrading indigestible fibers and polysaccharides (Liu et al., 2019; Zhang et al., 2019). Butyrate is a key fuel source for colonocytes, with an overall positive effect on the hindgut function of cows such as improved epithelial tight junctions and reduced inflammatory status (Oikonomou et al., 2013). Therefore, we speculated that a greater abundance of VFA producing bacteria and a lower abundance of pathogenic bacteria in FR and SR cows would not only benefit hindgut health but also liver metabolism.

As a primary bile acid, glycocholic acid was produced in hepatocytes, stored in the gallbladder, excreted to the intestine, reabsorbed into the blood, and resynthesized into primary bile acid in the liver. A previous study showed that the increased levels of primary bile acid were related to liver injury, which results in hepatocyte release of the primary bile acid (Chiang and Ferrell, 2018). The decreased levels of GCA in the FR cows were observed in the current, indicating that FR did not harm liver function. Secondary bile acids are formed from the primary bile acids under the action of bacteria in the intestine. The lower levels of secondary bile acids, including GDCA, TCDCA, TLCA, and TUDCA, are probably due to the lower level of primary bile acid. Another possible reason was that the bacteria associated with primary bile acid biotransformation was decreased in the FR cows compared to the Con cows. A small population of intestinal species in the genus *Clostridium*, including *Clostridium scindens*, *Clostridium hiranonis*, *Clostridium hylemonae* (*Clostridium cluster XVIa*), and *Clostridium sordelli* (*Clostridium cluster XI*) is participating in secondary bile acids producing (Ridlon et al., 2006). Our guess was proved by the decreased relative abundance of OTU1039 from genus unclassified *o_Clostridiales* in FR cows and further proved by a positive correlation between unclassified *o_Clostridiales* and TLCA.

Compared to SR and Con cows, we did not observe the difference in bile acids, but we found some difference in tryptophan metabolism. Tryptophan is taken up in the small intestine, but the fraction that reaches the colon can be catabolized by the gut bacteria resulting in a variety of indole-derivatives, such as IAA. Several *Bacteroides* species, as well as *Clostridium bartlettii* have been reported to produce IAA (Russell et al., 2013). The lower level of IAA might be attributed to the reduction of OTU1367 from genus unclassified *o_Clostridiales.* We also observed the increased abundance of OTU457 from genera unclassified *f_Ruminococcaceae* both in the FR group and SR group, which was negatively correlated with the relative concentration of methionine. unclassified *f_Ruminococcaceae* belonged to the family *Ruminococcaceae*, which is related to amino acid metabolism (Zhang et al., 2018). Thus, a higher relative abundance of unclassified *f_Ruminococcaceae* leads to a lower relative concentration of methionine in the serum.

Moreover, we also observed the relative abundance of OTU from genera *Ruminococcaceae* UCG-005 within the family *Ruminococcaceae* was positively correlated with the relative concentration of linoleic acid, indicating that *Ruminococcaceae* UCG-005 may play an important role in lipid metabolism. Consistent with this finding, in the study of chickens, *Ruminococcaceae* displayed a positive correlation with lipid metabolism (Cui et al., 2021). The elevation concentration of serum non-esterified fatty acid composition (palmitic acid and linoleic acid) was the outcome of increased lipolysis during the transition period, supporting that the reduced serum proportions of palmitic acid and linoleic acid in FR and SR cows may represent a favorable profile to inhibited lipolysis.

## Conclusion

In summary, the results of this study provide new evidence for an alteration of the fecal microbiota and host metabolism in fresh dairy cows after RFT. Different serum metabolites and bacterial communities at the OTU level were observed after RFT. The proportion of OTU1517 from genera *Ruminococcaceae* UCG-005 was dramatically decreased in both FR and SR cows. Moreover, RFT affected the amino acids, fatty acids and bile acids metabolism. Furthermore, correlation analysis showed a strong correlation between fecal bacteria and serum metabolites participating in tryptophan metabolism, bile acid metabolism and fatty acid metabolism, thereby reflecting that RFT not only affects the fecal microbiota but also influences the host metabolism. This work will provide a comprehensive understanding in assessing the effects of rumen fluid intervention in dairy cows and facilitate rumen microbial intervention on dairy performance improving and homeostasis restoring.

## Data Availability Statement

The datasets presented in this study can be found in online repositories. The names of the repository/repositories and accession number(s) can be found in the article/Supplementary Material.

## Ethics Statement

The animal study was reviewed and approved by Institutional Experimental Animal Care and Use Committee of the Ministry of Agriculture and Rural Affairs of China and the Animal Care and Use Committee at China Agricultural University.

## Author Contributions

SH and SL conceived and designed the study. SH and HM collected all samples used in this study. SH performed the data analysis and wrote the manuscript with contributions from WW. SH and GZ reviewed and revised the manuscript. SH and SL provided the funding. All authors read and approved the final manuscript.

## Conflict of Interest

The authors declare that the research was conducted in the absence of any commercial or financial relationships that could be construed as a potential conflict of interest.

## Publisher’s Note

All claims expressed in this article are solely those of the authors and do not necessarily represent those of their affiliated organizations, or those of the publisher, the editors and the reviewers. Any product that may be evaluated in this article, or claim that may be made by its manufacturer, is not guaranteed or endorsed by the publisher.

## Footnotes

1
http://proteowizard.sourceforge.net

2
http://www.genome.jp/kegg/

3
http://www.nist.gov/index.html

4
http://www.metaboanalyst.ca

5 https://cran.r-project.org/package/dplyr; author, H Wickham, R François, L Henry, K Müller; published date, 2018.

6 http://cran.r-project.org/web/packages/psych; author, W Revelle; published date, 2016.

## Supplementary Material

Supplementary Figure S1 The rarefaction curves for each fecal sample were collected. The smaller number of new OTUs increased as the sequence number increased, indicating that our sampling depth was adequate to cover the fecal microbial composition we tested.

Supplementary Figure S2 Box-and-whisker plots of alpha diversity indices (Chao1 and Shannon) for fecal microbial communities in saline (Con, n=8), fresh rumen fluid (FR, n=8) and sterilized rumen fluid (SR, n=8) cows. Kruskal-Wallis was performed to analyze alpha diversity indices difference across all groups.

Supplementary Figure S3 The dynamic distributions of fecal microbiota at phylum (A) and genus (B) levels. The IDs on the X-axis represent the samples from Con, FR and SR groups. C = saline, Con group; FR = fresh rumen fluid; SR = sterilized rumen fluid.

Supplementary Figure S4 Partial least squares-discriminate analysis (PLS-DA) scores plot between Con and FR, Con and SR, FR and SR groups based on LC/MS. Con = saline, FR = fresh rumen fluid, SR = sterilized rumen fluid.

## Abbreviations

ADF: acid detergent fiber
Con: control
DDGS: dried distillers grains with soluble
FR: fresh rumen fluid
GCA: glycocholic acid
GDCA: glycodeoxycholic acid
IAA: indoleacetic acid
NDF: neutral detergent fiber
OTUs: operational taxonomic units
PCA: principal component analysis
PCoA: principal coordinate analysis
PLS-DA: projections to latent structures discriminant analysis
RFT: rumen fluid transplantation
SR: sterilized rumen fluid
TCDCA: taurochenodeoxycholate
TLCA: taurolithocholic acid
TMAO: trimethylamine N-oxide
TUDCA: tauroursodeoxycholic
VIP: variable importance in projection.
