# Supplementary material for: The Response of Fecal Microbiota and Host Metabolome in Dairy Cows Following Rumen Fluid Transplantation
Source: Front Microbiol. 2022 Jul 13;13:940158. doi: 10.3389/fmicb.2022.940158 (PMC9343124; doi:10.3389/fmicb.2022.940158)
Supplement: Supplementary file 1 [file Table_1.DOCX]

**Table S1.** The chemical and nutritional components of the experimental diets.

| **Items^1^** | **Fresh** | **Early** |
| --- | --- | --- |
| **Ingredients**, % of dry matter basis |  |  |
| Alfalfa hay | 20.00 | 11.72 |
| Oat hay | 4.21 | 2.73 |
| Corn silage | 21.05 | 26.04 |
| Steam-flaked corn | 19.88 | 18.45 |
| Yeast Culture XP | 1.17 | 0.43 |
| Soybean meal | 20.23 | 17.80 |
| Cottonseed | 5.32 | 7.90 |
| Molasses | 2.28 | 2.69 |
| Ground corn | — | 3.91 |
| DDGS | — | 1.95 |
| Sprayed corn hull | — | 1.17 |
| 5% premix^2^ | 3.80 | 2.82 |
| BergaFat | 1.46 | 1.74 |
| Sodium bicarbonate | 0.58 | 0.65 |
| **Contents**, %^3^ |  |  |
| Crude protein | 18.08 | 17.41 |
| Ether extract | 5.04 | 5.64 |
| ADF | 20.65 | 19.89 |
| NDF | 30.40 | 28.54 |
| NE_L_, Mcal/kg | 1.78 | 1.82 |
| Ca | 0.97 | 0.78 |
| P | 0.47 | 0.43 |

^1^DDGS dried distillers grains with solubles, NDF neutral detergent fiber, ADF acid detergent fiber, Ca calcium, P phosphorus.

^2^The 5% premix was composed by limestone, calcium hydrophosphate, sulfate manganese, calcium, zinc, copper, sodium chloride, magnesium oxide, magnesium sulfate, vitamin A, D3 and E, sodium selenite, calcium phosphate, cobalt chloride and saccharomyces cerevisiae, et al.

^3^NEL: nutrient contents were measured value and NEL was calculated value with model from NRC (2001).
