# Supplementary figures and images for: The Response of Fecal Microbiota and Host Metabolome in Dairy Cows Following Rumen Fluid Transplantation
Source: Front Microbiol. 2022 Jul 13;13:940158. doi: 10.3389/fmicb.2022.940158 (PMC9343124; doi:10.3389/fmicb.2022.940158)

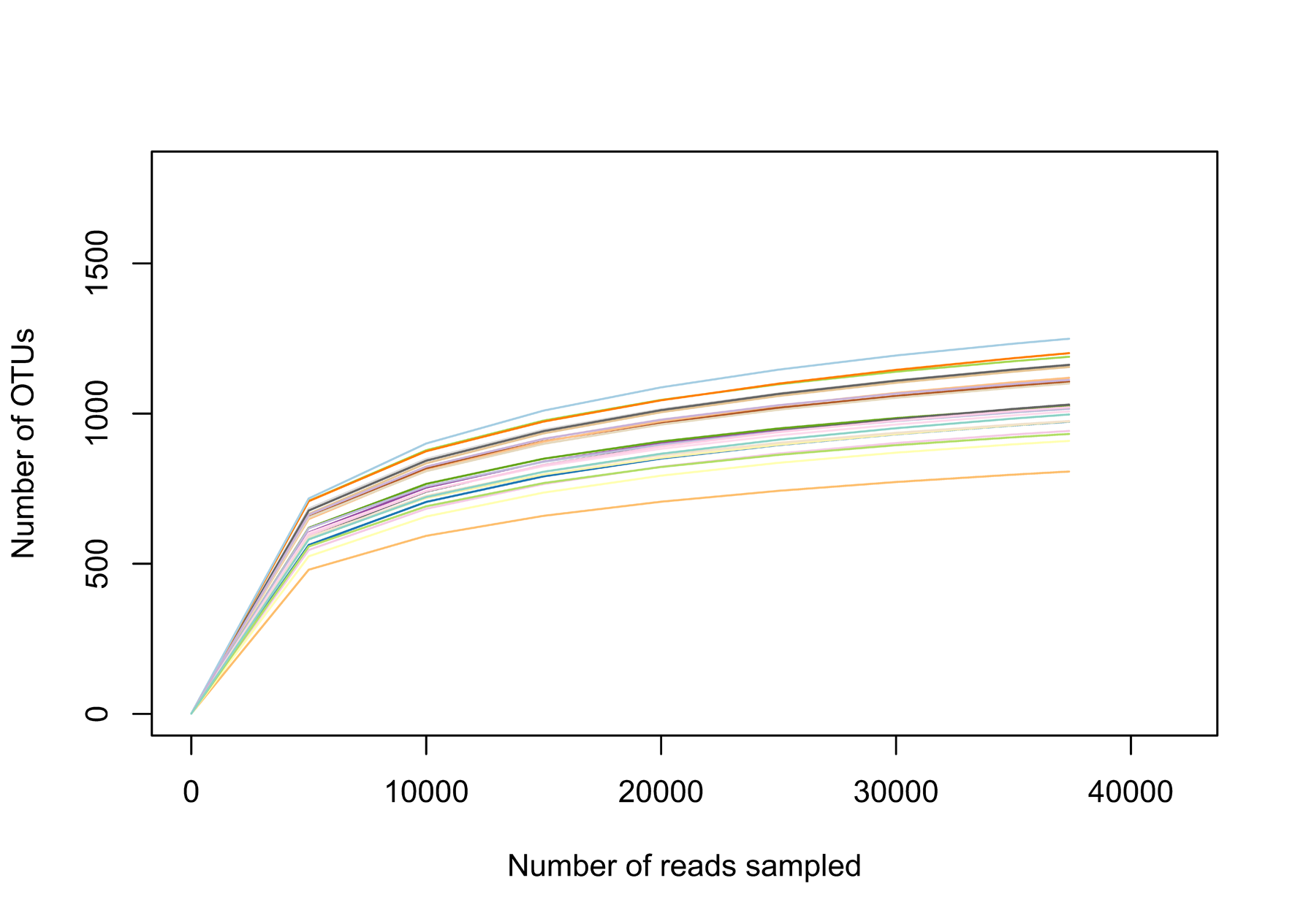

Supplement: Supplementary Figure S1 — The rarefaction curves for each fecal sample were collected. The smaller number of new OTUs increased as the sequence number increased, indicating that our sampling depth was adequate to cover the fecal microbial composition we tested. [file Image_1.tiff]

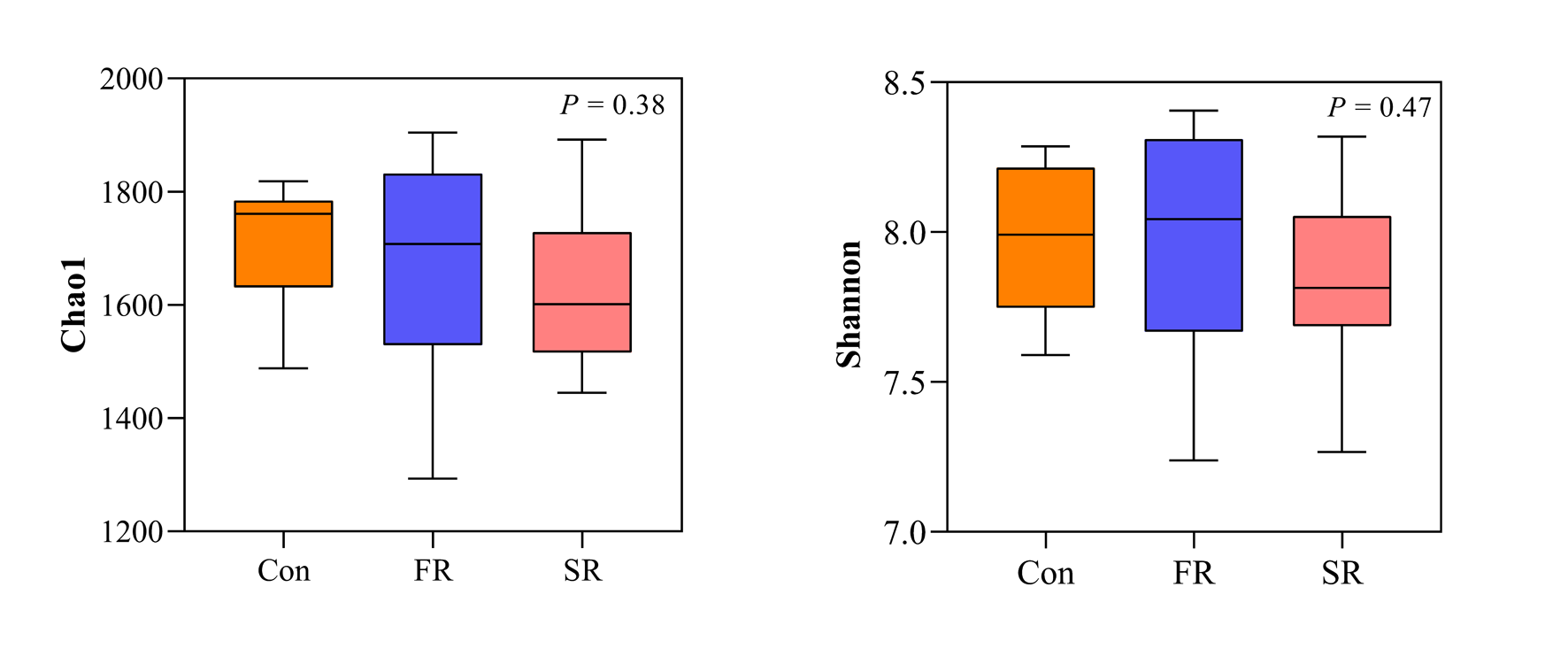

Supplement: Supplementary Figure S2 — Box-and-whisker plots of alpha diversity indices (Chao1 and Shannon) for fecal microbial communities in saline (Con, n=8), fresh rumen fluid (FR, n=8) and sterilized rumen fluid (SR, n=8) cows. Kruskal-Wallis was performed to analyze alpha diversity indices difference across all groups. [file Image_2.tiff]

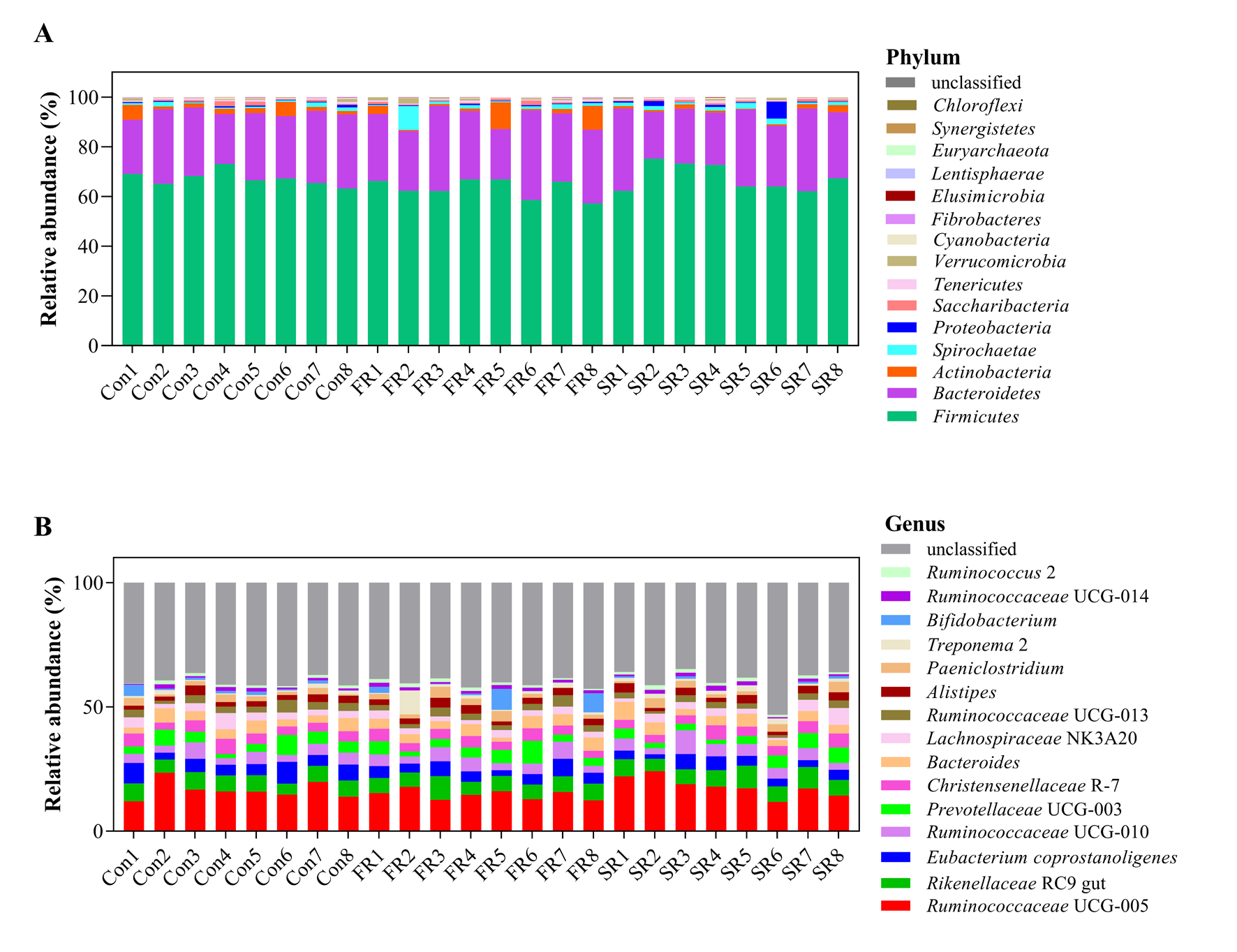

Supplement: Supplementary Figure S3 — The dynamic distributions of fecal microbiota at phylum (A) and genus (B) levels. The IDs on the X-axis represent the samples from Con, FR and SR groups. C = saline, Con group; FR = fresh rumen fluid; SR = sterilized rumen fluid. [file Image_3.tif]

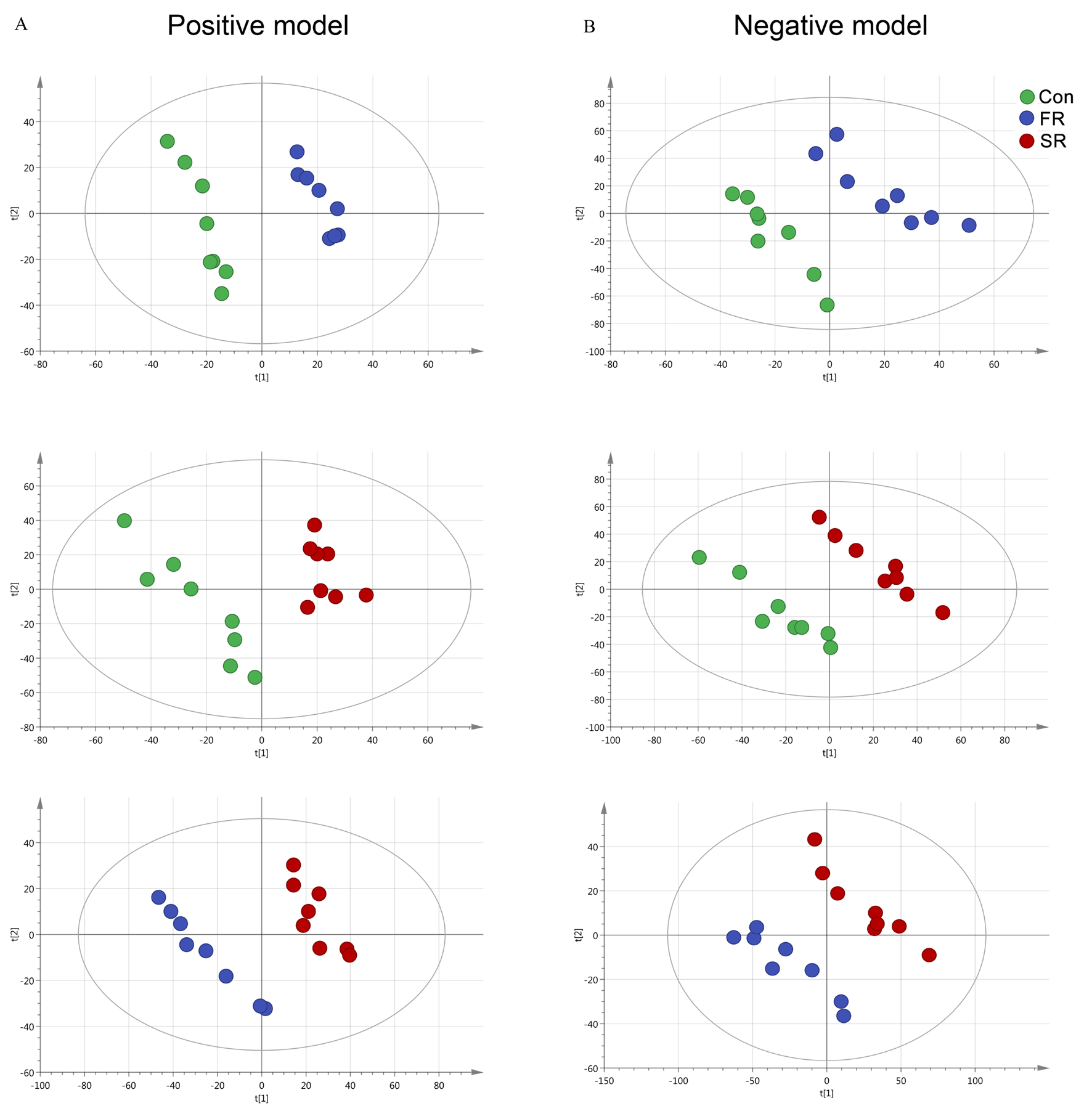

Supplement: Supplementary Figure S4 — Partial least squares-discriminate analysis (PLS-DA) scores plot between Con and FR, Con and SR, FR and SR groups based on LC/MS. Con = saline, FR = fresh rumen fluid, SR = sterilized rumen fluid. [file Image_4.tiff]
